# The Effects of Paroxetine on Benthic Microbial Food Web and Nitrogen Transformation in River Sediments

**DOI:** 10.3390/ijerph192114602

**Published:** 2022-11-07

**Authors:** Yi Li, Xinqi Chen, Xinzi Wang, Jiahui Shang, Lihua Niu, Longfei Wang, Huanjun Zhang, Wenlong Zhang

**Affiliations:** 1 Key Laboratory of Integrated Regulation and Resource Development of Shallow Lakes of Ministry of Education, College of Environment, Hohai University, Nanjing 210098, China; 2 Jiangsu Nanjing Environmental Monitoring Center, Nanjing 210013, China

**Keywords:** paroxetine, microbial food web, nitrogen, DNA metabarcoding, microbenthic communities

## Abstract

Paroxetine is a common pharmaceutical to treat depression and has been found to pose threats to aquatic organisms. However, little is known about the effects of paroxetine on the nutrient cycle in aquatic environments. Therefore, DNA metabarcoding is used in this study to analyze the effects of paroxetine on multi-trophic microorganisms and nitrogen transformation in river sediments. Although paroxetine has no significant effect on the diversity of microbenthos, changes in benthic nitrogen-converting bacteria are consistent with the change in the various forms of nitrogen in the sediment, indicating that paroxetine affects the nitrogen conversion process by affecting nitrogen-converting bacteria. In addition, it is found that paroxetine has the ability to influence nitrogen transformation in an indirect way by affecting the trophic transfer efficiency of higher trophic levels (meiofauna and protozoa, protozoa and protozoa), subsequently affecting the growth of nitrogen-converting bacteria through a top-down mechanism (i.e., predation).The results show that paroxetine affects nitrogen transformation directly by affecting nitrogen-converting bacteria and indirectly through top-down effects, emphasizing that the assessment of paroxetine’s ecological risks should consider species within different trophic levels.

## 1. Introduction

Recently, the incidence of depression, a type of mental illness, has been on the rise, possibly owing to the 2019 coronavirus disease (COVID-19) pandemic, leading to an increase in the use of antidepressants [1,2,3]. Antidepressant compounds have been continuously released into aquatic environments due to their overuse and careless discarding, as well as insufficient removal from wastewater treatment systems [4,5]. Antidepressant concentrations have been detected ranging from ng/L to μg/L in North American and European surface waters and sediments [6]. Notably, concentrations of antidepressants have been found to reach 0.509 μg/L in a Canadian sewage treatment plant [7]. While relatively low concentrations of compounds are found in aquatic environments, their high targeted bioactivity and metabolic stability make them a threat to aquatic organisms and even aquatic ecosystems once they enter into a water body [6,8,9]. With the increasing demand for antidepressants to alleviate depressive disorders, concentrations of antidepressants are likely to increase in aquatic environments, calling for scientific investigations of their influence on ecosystems.

Selective serotonin reuptake inhibitors (SSRIs) are currently one of the most widely used antidepressants to treat depression [5,10]. Serotonin is an important neurotransmitter found in a variety of living organisms and participates in many physiological processes, such as metabolism, growth, and reproduction [4,11]. As a result, the addition of SSRIs to aquatic environments probably has negative effects on both physiological and neuronal processes in aquatic organisms by regulating serotonin or interfering with enzyme metabolism. For example, Vaclavik et al. [11] found that SSRIs had a significant negative effect on fish behavior, leading to inhibition of the escape reflex and enhancement of the anti-stress ability in fish. Zhu et al. [12] have noted that SSRIs harm the survival characteristics of daphnia species by changing the predation relationship. Many studies to date have focused on the ecological effects of SSRIs on aquatic macroorganisms and less on microbenthos. SSRIs are easy to accumulate in sediments considering the degradation resistance of antidepressants and the adsorption by sediments [13,14]. Microbenthic organisms, a significant part of aquatic benthic environments, participate in organic matter degradation and nutrient cycling. Additionally, SSRIs can be bioaccumulated and biomagnified through the food chain or food web, thus posing threats to aquatic ecosystems through bottom-up and top-down effects [15]. Therefore, investigations are presently needed regarding the effects of SSRIs on microbenthos and the microbial food web.

Nitrogen is an essential nutrient for many living organisms, and its cycling is an important component of biogeochemical cycling. Once nitrogen cycling is out of balance, a series of ecological and environmental problems can be caused, such as eutrophication, hypoxia, and an increase in greenhouse gas emissions [16,17]. Aquatic sediment is the media enabling many nitrogen transformations, such as nitrification and denitrification [18]. Nitrogen distribution in benthic environments has been found to be affected by inputs and outputs of various organic pollutants [19,20,21,22,23]. SSRIs are a new type of persistent organic pollutants, and hence their effects on benthic nitrogen transformation also require investigation. In addition, bacteria play an essential role in nitrogen transformation, including organic nitrogen degradation and inorganic nitrogen compound transformation [24]. Importantly, bacteria do not exist in isolation in aquatic ecosystems and are affected by top-down and bottom-up effects in the food web. However, the role of other trophic microbenthos is largely neglected in many studies that investigate the effect of organic pollutants on nitrogen transformation. Thus, it is necessary to investigate the effects of antidepressants on both microbenthos and nitrogen transformation in aquatic ecosystems from a food web perspective.

Given the knowledge gaps described above, the aim of this study is to answer the following questions: (i) What are the effects of SSRI antidepressants on nitrogen transformation in sediments? (ii) What are the effects of SSRI antidepressants on aquatic microbenthos, including bacteria, protozoans and metazoans? (iii) How do benthic microbial food webs relate to nitrogen transformation following the addition of SSRI antidepressants? To answer these questions, paroxetine was selected as a typical SSRI antidepressant, and DNA metabarcoding was used to describe the effects of paroxetine on multi-trophic species and nitrogen transformation in sediments. The results of this study will help to reveal the nitrogen transformation process under the addition of SSRI antidepressants in water bodies and to evaluate the potential harm caused by SSRI antidepressants to benthic multi-trophic microorganisms, providing a theoretical and scientific basis for the improved management of aquatic environments.

## 2. Materials and Methods

### 2.1. Study Area and Sample Collection

A sample collection was conducted in March 2019 at the Sancha Estuary of the Yangtze River, located at 32.07° N and 118.73° E. After testing, paroxetine was not detected in the collected samples. A Petersen grab sampler was used to randomly collect 0–15 cm of surface sediments, and above the sampling sites, in situ water samples were collected. Samples of water and sediment were collected in polyethylene bottles, stored in ice boxes, and transported back to the laboratory. The sediment samples were thoroughly mixed after screening out large debris using a 2 mm sieve. Each mixed sample was equally divided into two parts: one was stored at −80 °C for DNA extraction, and the rest was stored at 4 °C prior to the microcosm experiments.

### 2.2. Microcosm Experiments and Environmental Parameter Analysis

A standard test solution was prepared by dissolving paroxetine (CAS: 110429-35-1) in methanol. After that, it was diluted to 10 μg/L (close to its environmental concentrations), 100 μg/L (close to microbenthos’ lowest-observable effect concentration), and 1000 ug/L (close to the lowest-observable effect concentrations for macrobenthos).

An aerobic sediment microecosystem was prepared consisting of 0.2 L water and 0.1 L sediment. Four different treatment groups were established by adding paroxetine to the sediment: (A) Control group (no paroxetine added); (B) Low concentration paroxetine-treatment group (addition of 10 μg/L paroxetine); (C) Moderate concentration paroxetine-treatment group (addition of 100 μg/L paroxetine); (D) High concentration paroxetine-treatment group (addition of 1000 μg/L paroxetine). Nine replicates of each treatment were used to study the changes in multitrophic microbenthos. Breathable sealing films were used to maintain aerobic conditions. To simulate natural conditions, the microcosms were illuminated for 16 h a day at 20 °C. The incubation time was 18 days.

Sediments were sampled on days 0, 6, 12, and 18. Samples were divided into two parts. One was used to measure environmental parameters, and the remainder was stored at 4 °C before DNA sequencing. Concentrations of NO_3_-N, NO_2_-N and NH_3_-N in the sediments were determined according to the national standard method, as described by Li et al. [9].

### 2.3. eDNA Extraction, PCR Amplification, and Sequencing Analysis

Microbial DNA was extracted from surface sediment using the Power Soil DNA Isolation Kit (Mo Bio Laboratories Inc., Carlsbad, CA, USA). Agar gel electrophoresis was used to identify the integrity of the extracted DNA. The V3–V4 region of the 16S rRNA gene was then amplified using primers 341F and 806R. Analysis of eukaryotic sequences was conducted through amplification of the 380-bp fragment of the 18S rRNA V4 region. In this process, TAReuk454FWD1 and TAReukREV3 were used as eukaryotic primers. PCR used TransStart Fastpfu DNA polymerase. The 20 μL reaction system consisted of 4 μL 5 × FastPfu Buffe, 2 μL 2.5 mM dNTPs, 0.8 μL forward primer (5 μM), 0.8 μL reverse primer (5 μM), 0.4 μL FastPfu polymerase and 10 ng template DNA. The reaction process was conducted according to protocols as described in [9].

Readings were discarded if they contained no primers, and sequences were discarded if they contained uncalled bases. Analysis and processing of the remaining sequencing reads and generation of Fasta files were performed with QIME V.1.9.1. The UCLUST clustering method was used to cluster OTUs with a 97% identity threshold. The SILVA 128 database and SILVA 132 database were used to compare representative OTUs with database records to obtain the final OTU table for prokaryotes and eukaryotes, respectively.

### 2.4. Statistical Analysis

Significantly differentiated species were obtained by using LDA Effect Size (LEfSe) analysis at a threshold of 3.0, available at the website http://huttenhower.sph.harvard.edu/lefse/ (accessed on 17 May 2021). Phylogenetic Investigation of Communities by Reconstruction of Unobserved States (PICRUSt) function prediction was conducted to better acquire functional information in the benthic microbial communities. Alpha diversity (α-diversity), including the Chao1 index, ACE index, Shannon index, and Simpson index, was used to analyze the diversity of the eukaryotic communities. Principal component analysis (PCA) was used to describe differences in community composition between samples.

To illustrate characteristics of the microbial food web, OTU numbers were used as biomass to calculate trophic transfer efficiencies between species at different trophic levels. In addition, a structural equation model (SEM) was used to explore the top-down effect in the obtained microbial food web, analyze the correlation between species at multiple trophic levels, and calculate pathways between trophic transfer efficiency and nitrogen concentration under the addition of paroxetine. The SEM model was established using IBM SPSS AMOS (Version 22).

Some indexes, including the comparative fitting index (CFI), chi-square degree of freedom ratio (CMIN/DF (χ^2^/DF)), and incremental fitting index (IFI), were calculated to evaluate the fitting degree of the model. Heterotrophic flagellates (HF): bacteria, ciliates: bacteria, and amoeba: bacteria were identified as the nutrient transfer efficiency from protozoa to bacteria. Ciliates: HF was identified as the nutrient transfer efficiency within the protozoa. The nutrient transfer efficiency between meiofauna and bacteria was calculated as meiofauna: bacteria. The nutrient transfer efficiency from meiofauna to protozoa was calculated as meiofauna: protozoa. Note that HF, amoeba, and ciliates belonged to the protozoa.

## 3. Results and Discussion

### 3.1. Effect of Paroxetine on Different Forms of Nitrogen in Sediments

**Figure 1:**
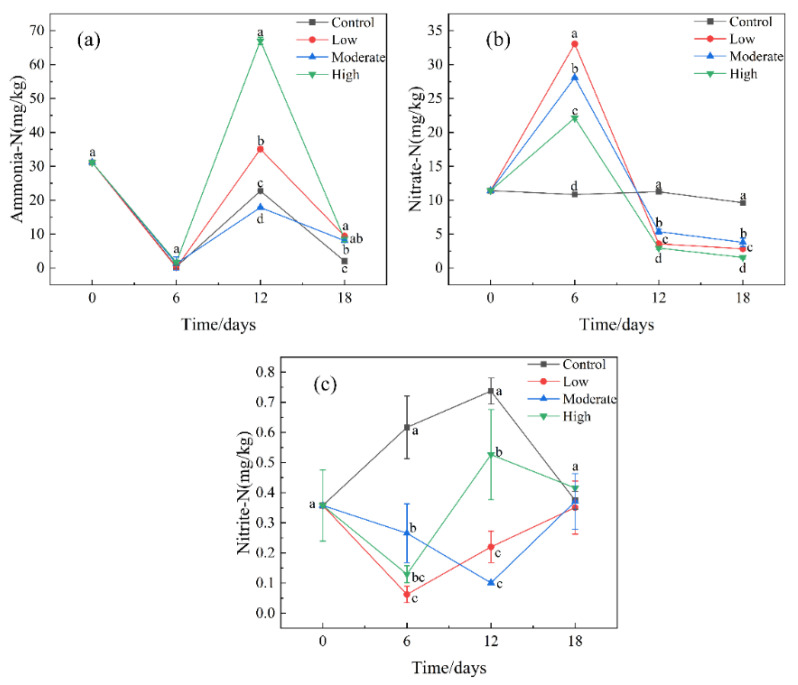
Changes of (**a**) Ammonia-N, (**b**) Nitrate-N, and (**c**) Nitrite-N on day 0, 6, 12, and 18 in all treatment groups. Control: the sediment with no paroxetine added; Low: 10 μg/L paroxetine; Moderate: 100 μg/L paroxetine; High: 1000 μg/L paroxetine. The error bars represent the mean value of three groups of these samples, and the letter represents significant differences (*p* < 0.05).

Figure 1 shows the changes in the various forms of nitrogen in the sediment under different paroxetine concentrations. On day 6, the contents of nitrate in the paroxetine-treated groups (22.1–33.1 mg/kg) were significantly higher than in the control group (10.8 mg/kg). Paroxetine significantly promoted nitrate accumulation in the sediments (*p* < 0.05). On day 12, the nitrate concentrations (3.0–5.4 mg/kg) in the paroxetine-treated groups were lower than those in the control group (11.3 mg/kg). At the late stage of culture (18 days), nitrate concentrations in the treatment groups continued to decrease (1.6–3.8 mg/kg), and nitrate concentration in the high-concentration treatment group was the lowest, which indicated that a high concentration of paroxetine had the most significant inhibition on accumulating nitrate concentration.

### 3.2. Effect of Paroxetine on Bacterial Communities in Sediment

**Figure 2:**
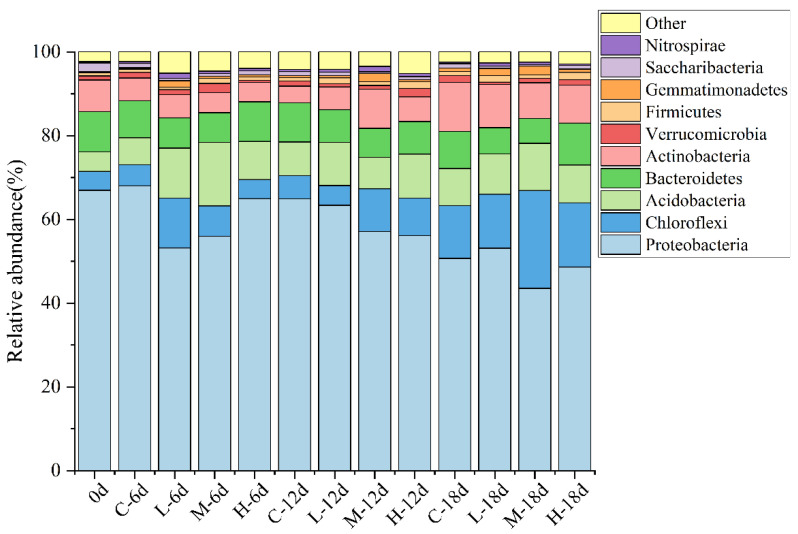
Relative abundance of bacteria communities at the phylum level under different times. C: the sediment with no paroxetine added; L: 10 μg/L paroxetine; M: 100 μg/L paroxetine; H: 1000 μg/L paroxetine.

The relative abundance of bacterial communities at the phylum level at different times is shown in Figure 2. Proteobacteria was the dominant bacterial phylum, accounting for 44% to 68%. On day 6, compared with the control group, paroxetine had an inhibitory effect on Proteobacteria. After 12 days of paroxetine treatment, the relative abundance of Proteobacteria in the treatment groups was still lower than that in the control group. As the paroxetine concentration increased, the inhibition of paroxetine on Proteobacteria growth became more prominent. On day 18, low-concentration paroxetine stimulated Proteobacteria, but moderate and high concentrations of paroxetine still inhibited Proteobacteria growth. Other dominant bacterial phyla included Chloroflexi (5–23%), Acidobacteria (5–15%), Bacteroidetes (6–10%), Actinobacteria (4–12%), Verrucomicrobia (1–2%), Firmicutes (1–2%), Gemmatimonadetes (1–2%), Saccharibacteria (1–2%), Nitrospirae, and one phylum without classification. Proteobacteria have been shown to have the ability to fix nitrogen [25]. However, the changes in Proteobacteria were not in conjunction with the observed changes in ammonia concentration. Considering that functional microorganisms ultimately determine the nitrogen transformation performance, it is necessary to identify whether there were bacterial taxa with significant abundance differences between the paroxetine-treated and control groups and compare the functional bacteria of the two groups.

**Figure 3:**
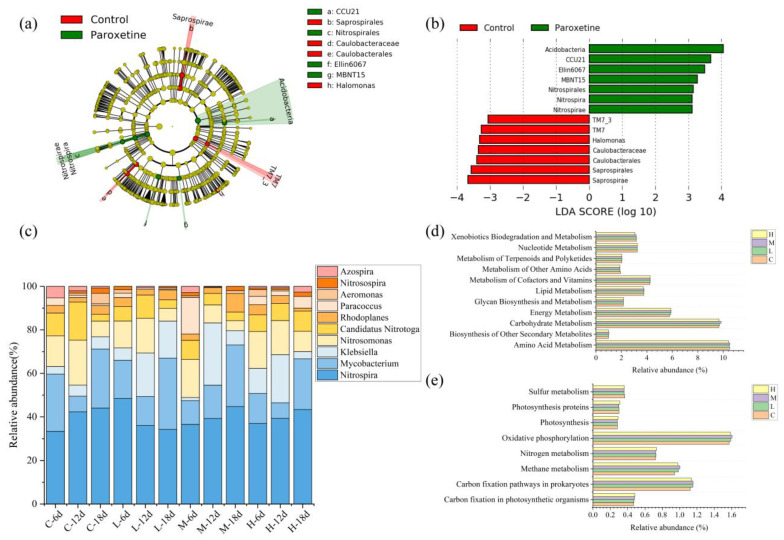
Bacterial taxa with significant abundance differences between control and paroxetine groups and the relative abundance of nitrogen-transforming bacteria and predicted functional profiles in different treatment groups. (**a**) is the cladogram of bacterial communities. (**b**) is LDA score identified the size of differentiation between the control and paroxetine groups with a threshold value of 3.0. (**c**) is the relative of nitrogen-transforming bacteria in different treatment groups. (**d**) is the relative abundance of metabolism in KEGG categories (level 2). (**e**) is the relative abundance of energy metabolism in KEGG categories (level 3). C: the sediment with no paroxetine added; L: 10 μg/L paroxetine; M: 100 μg/L paroxetine; H: 1000 μg/L paroxetine.

To determine bacterial taxa with significant abundance differences between the control group and paroxetine-treated groups, the linear discriminant analysis (LDA) effect size (LEfSe) method was used to perform biomarker analysis. As shown in Figure 3a,b, 14 bacterial clades presented statistically significant differences with an LDA threshold of 3.0. TM7_3, TM7, Halomonas, Caulobacteraceae, Caulobacterales, Saprospirales, and Saprospirae were enriched in the control group. In the paroxetine-treated groups, Acidobacteria, CCU21, Ellin6067, MBNT15, Nitrospirales, Nitrospira, and Nitrospirae were enriched. Nitrospira has been identified as a key functional group to perform nitrification [26]. The results of LEfSe showed that the presence of paroxetine might affect functional bacteria involved in nitrification in sediments and thus affect the process of nitrogen transformation.

Based on the FAPROTAX database, the top 10 bacteria at the genus level involved in nitrogen transformation were screened out to observe the effects of different concentrations of paroxetine on nitrogen-transforming bacteria (Figure 3c). On day 6, the relative abundance of Nitrospira in the paroxetine group was higher than that in the control group. Nitrospira has been identified as complete ammonia oxidizing (commamox) bacteria, which completely oxidize ammonia to nitrate [27], and hence may compete with aerobic ammonia oxidizing bacteria (AOB) and nitrite oxidizing bacteria (NOB) [28]. This meant that in the early stages, Nitrospira is converted directly from ammonia nitrogen to nitrate nitrogen, leading to a decrease in the content of ammonia nitrogen and an increase in the content of nitrate nitrogen in the paroxetine group (Figure 1). In addition, the relative abundance of Mycobacterium in the paroxetine group is lower than that in the control group. Mycobacterium have been found to metabolize nitrate during aerobic growth [29,30], which meant that paroxetine inhibited Mycobacterium from converting nitrate nitrogen into nitrite nitrogen. Thus, the content of nitrite decreased (Figure 1). On day 12, the relative abundance of Nitrospira in the paroxetine-treated groups was lower than that in the control group, meaning that paroxetine began to inhibit the commamox bacteria from forming nitrate and the concentration of nitrate nitrogen decreased (Figure 1). In addition, Figure 3c shows the relative abundance of Klebsiella in paroxetine-treated groups on day 12 was higher than that on day 6. Klebsiella have been proved to carry out nitrogen assimilation by using nitrate as an nitrogen source for growth and reducing nitrate to ammonia nitrogen via nitrite [31]. Therefore, Klebsiella in the paroxetine-treated group was promoted to convert nitrate to nitrite and further to ammonia nitrogen on day 12, resulting in the accumulation of ammonia nitrogen. At the late stage of culture (18 days), paroxetine blocked the process of oxidizing ammonia to nitrate by inhibiting Nitrospira, so that the content of nitrate nitrogen decreased and the content of ammonia nitrogen accumulated (Figure 1). In addition, compared with the control group, low and moderate concentrations of paroxetine promoted the growth of Mycobacterium, while high concentrations of paroxetine inhibited it. On day 18, dissolved oxygen was gradually depleted. Mycobacterium can reduce nitrate to nitrite under anaerobic conditions [32]. Therefore, on day 18, the nitrite nitrogen in the low and moderate paroxetine-treated groups increased, while the nitrite nitrogen in the high paroxetine-treated group decreased (Figure 1).

Figure 3d,e shows the metabolism pathways of bacterial communities at different levels as predicted by PICRUSt. Functions related to paroxetine treatment in level 1 (Supplementary Figure S1) mainly included Environmental Information Processing (12.93–13.11%), Genetic Information Processing (16.61–16.69%), and Metabolism (49.31–49.76%). Metabolism occupied the highest relative abundance, and paroxetine had a positive effect on metabolism. Hence, the distribution of metabolism functions in level 2 (Figure 3d) was specifically analyzed. For metabolism, functions of Biosynthesis of Other Secondary Metabolites (0.99–1.01%), Carbohydrate Metabolism (9.68–9.84%), Energy Metabolism (5.87–5.90%), Glycan Biosynthesis and Metabolism (2.16–2.17%) were enriched in the paroxetine-treated groups.

In order to further explore the influence of paroxetine on eleven metabolism functions, a variance analysis was conducted on metabolism functions under the action of paroxetine (Supplementary Table S1). The results showed that paroxetine had significant differences in the biosynthesis of other secondary metabolites, carbohydrate metabolism, and carbohydrate metabolism (*p* < 0.05). This study mainly explored the effect of paroxetine on nitrogen metabolism and transformation. Therefore, the influence of paroxetine on energy metabolism was analyzed in detail (Figure 3e).

For energy metabolism, carbon fixation in photosynthetic organisms (0.47–0.48%), carbon fixation pathways in prokaryotes (1.14–1.15%), methane metabolism (0.98–1.00%), nitrogen metabolism (0.72–0.73%), oxidative phosphorylation (1.58–1.60%) were enriched under paroxetine treatment. Compared with the control group, paroxetine stimulated the function of nitrogen metabolism. Therefore, paroxetine might affect nitrogen transformation by stimulating the bacterial nitrogen metabolism function. Previous research has also shown that higher trophic levels like protozoa and meiofauna play strong regulating roles in microbiota [33]. Hence, future studies should focus on the effect of paroxetine on eukaryotes.

### 3.3. Effect of Paroxetine on Eukaryotic Communities in Sediments

**Figure 4:**
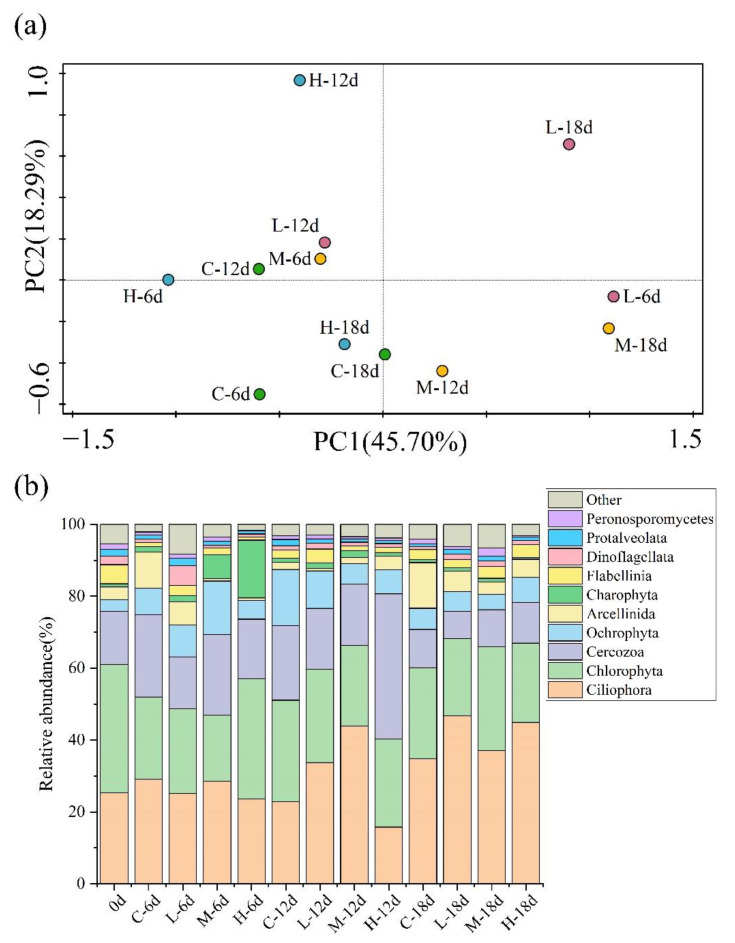
(**a**) PCA plot of eukaryotic communities at class level under different paroxetine-treated groups on day 6, day 12, and day 18. (**b**) Relative abundances of the eukaryotic communities over different time periods (at order level). C: the sediment with no paroxetine added; L: 10 μg/L paroxetine; M: 100 μg/L paroxetine; H: 1000 μg/L paroxetine.

By analyzing the α-diversity indices of eukaryotic microbial communities (Table 1), the species diversity and richness of eukaryotic communities were obtained. The results showed that the addition of paroxetine significantly increased the species richness of eukaryotes (Chao1 index and ACE index) on day 6 and showed a reverse trend at later stages. The addition of a high concentration of paroxetine (1000 μg/L) significantly affected eukaryotic species richness. In addition, only the addition of low paroxetine concentrations increased species diversity (Shannon index and Simpson index) at the initial stage (day 6), and paroxetine did not significantly affect species diversity at later stages. This showed that eukaryotic communities had a strong disturbance ability. Variance analysis results (Supplementary Table S2) showed that time gradient and concentration had no significant effect on the α-diversity index of eukaryotic communities. This result was consistent with previous studies, which found that antidepressants have little effect on reproductive and other physiological behaviors [34]. The result of PCA (Figure 4a) showed that there was a significant difference in community composition between the paroxetine-treated group and the control group. Changes in eukaryotic community structure are easily regulated by interactions in the food web [35], so paroxetine may affect eukaryotic community structure by influencing the interactions between trophic levels in the microbial food web.

Figure 4b shows the results of the analysis of the community composition of eukaryotes at the order level. The eukaryotic communities were dominated by Ciliophora (16–47%), followed by Chlorophyta (16–36%), Cercozoa (8–40%), and Ochrophyta (3–16%). As an important component of the aquatic environment ecosystem, protozoa had a high indication function for environmental pressure. As the most common protozoa, Ciliophora were stimulated by paroxetine. During the initial stage of culture (day 6), paroxetine had no obvious effect on Ciliophora. During the middle and late stages of culture, paroxetine with low and moderate concentrations had a significant impact on the growth of Ciliophora, while paroxetine with a high concentration showed a trend of initially inhibiting and then promoting the growth of Ciliophora. Ciliates display vital functional diversity as key elements of the microbial food web, acting as predators of bacteria and prey for meiofauna [36]. Previous studies have found that the grazing activities of ciliates can have a significant impact on regulating bacterial activity and community structure [37,38]. Ciliates have also been considered a controlling factor for nitrifying bacteria and nitrification [37]. Therefore, the change in the relative abundance of ciliates may affect the bacterial community, thus affecting the change in nitrogen concentration in the ecosystem.

Further analyses of protozoan communities’ distribution and composition at the class and order level are shown in Supplementary Figure S2a,b. At the class level, Alveolata, Holozoa, Chloroplastida, and Rhizaria were the dominant groups. At the order level, metazoa and ciliophora were the dominant groups. As the key parts of the microbial food web, protozoa and metazoa play an important role in material circulation and energy flow [39]. Heterotrophic flagellates (HF), amoeba, and Alveolata (ciliates) were the main predators of bacteria in protozoa. Large ciliates can also feed on flagellates and some small ciliates. As the main biological community in the benthic system, metazoa (meiofauna) occupied a high trophic level in the microbial food web.

**Figure 5:**
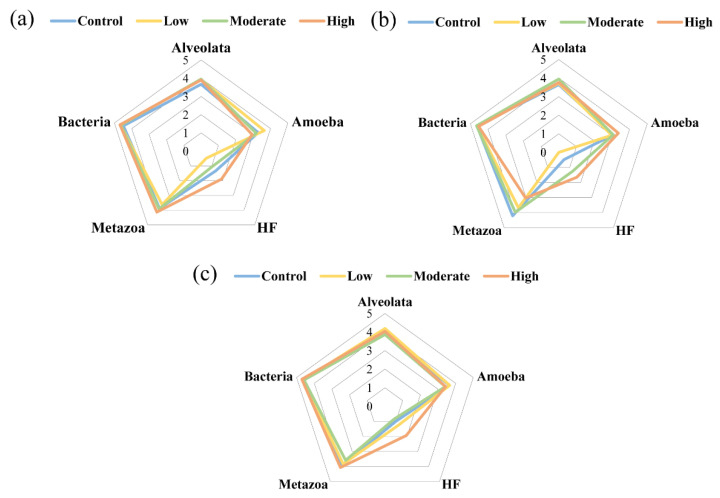
The main components in the obtained microbial food web during different periods. Logarithms (log10) taken for biomass reduce absolute errors. (**a**) Day 6; (**b**) Day 12; (**c**) Day 18. Control: the sediment with no paroxetine added; Low: 10 μg/L paroxetine; Moderate: 100 μg/L paroxetine; High: 1000 μg/L paroxetine.

Therefore, considering the biological composition of the actual microbial food web and the dominant biological groups, this study focused on HF, Alveolata (ciliates), amoeba, and metazoa (meiofauna) as the main parts in the microbial food web and analyzed the variances in biomass under the action of different concentrations of paroxetine (Figure 5). On day 6, high-concentration paroxetine slightly promoted the biomass of metazoa (Figure 5a). In the middle stage of culture (day 12), the biomass of metazoa decreased significantly (Figure 5b) and gradually recovered in the late stage of culture (day 18, Figure 5c). In addition, the biomass of HF was significantly promoted by the action of high-concentration paroxetine. However, there was no significant correlation between the biomass change of a single trophic level (protozoa and metazoa) and the nitrogen concentration change in the microbial food web (*p* > 0.05). This showed that the nitrogen transformation could not be analyzed based on a single trophic level. The experimental results further showed that paroxetine might affect the nitrogen transformation process by affecting the interaction among trophic levels of the microbial food web.

### 3.4. Top-Down Controls in the Microbial Food Web under the Influence of Paroxetine

**Figure 6:**
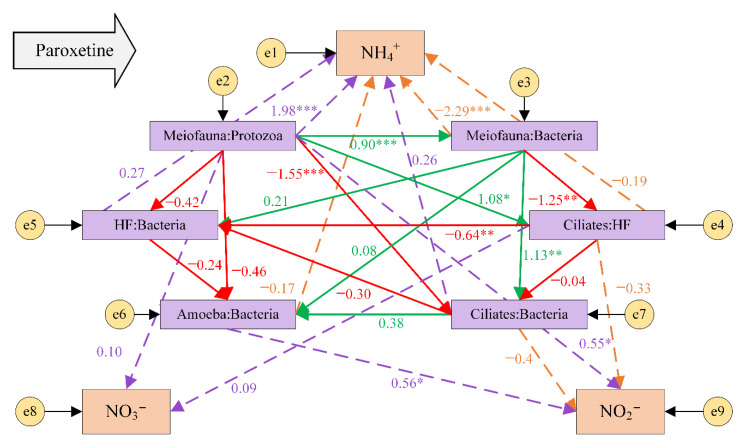
An analysis of the trophic transfer efficiency in response to the paroxetine addition using a structural equation model. HF are heterotrophic flagellates. Correlations are represented by different colors. Green and purple represent positive correlations; red and orange represent negative correlations; standardized path coefficients represented by numbers are close to the arrows. * indicates different degrees of significance (* *p* < 0.10; ** *p* < 0.05; *** *p* < 0.01).

Using the SEM, the causal relationship between the trophic transfer efficiency and nitrogen concentration change in the obtained microbial food web under the action of paroxetine was further explored, and how the interaction between multiple trophic levels affected nitrogen conversion was clarified (Figure 6). The goodness-of-fit indices, CMIN/DF (χ^2^/DF) = 1.425 (1–3), CFI = 0.902 (>0.90), IFI = 0.939 (>0.90), indicated that this model fitted reasonably well. Supplementary Table S3 presents the calculated results of all variables’ direct, indirect, and total influences.

There was a negative correlation between meiofauna: protozoa and ciliates: bacteria (r = −1.55, *p* = 0.003). The correlation between ciliates: HF and HF: Bacteria (r = −0.64, *p* = 0.023) had similar results. This suggests that protozoa such as ciliates, as the second trophic level in the microbial food web, connected the channel between meiofauna and Bacteria. Meiofauna: protozoa had a positive correlation with meiofauna: Bacteria (r = 0.90, *p* < 0.001), which indicates that the food for meiofauna was diverse and the microbial food web could be still stable under the interference of paroxetine.

Trophic-level interaction showed 73% of the change in the content of ammonia concentration and 42% of the change in the content of nitrite concentration. Meiofauna: protozoa was positively correlated with the change of ammonia nitrogen (r = 1.98, *p* < 0.001), while meiofauna: bacteria was negatively correlated with the change of ammonia nitrogen (r = −2.29, *p* < 0.001), indicating that bacteria, as the main participant in nitrogen transformation, were also regulated by the higher trophic levels.

The change of nitrate concentration was directly related to ciliates: HF and meiofauna: Protozoa. This suggests that paroxetine mainly affected the trophic transfer efficiency of the higher trophic levels and then affected nitrogen-transforming bacteria through top-down action in the microbial food web, thus affecting nitrogen transformation. Thus, the analyses showed that at the initial stage of paroxetine input, paroxetine promoted predation between higher trophic levels (predation of protozoa by meiofauna and predation between protozoa) so that more bacteria had the opportunity to perform nitrogen metabolism and nitrate concentration in sediments increased. In the middle and late stages of culture (days 12–18), paroxetine began to inhibit the predation between higher trophic levels and promote the predation of protozoa on bacteria, so that the process of bacteria converting ammonia nitrogen to nitrate nitrogen was inhibited and the content of nitrate nitrogen decreased, leading to ammonia nitrogen accumulating in river sediments. The results further confirm that protozoa and meiofauna play an important role in material circulation. Thus, it is necessary to analyze the effect of paroxetine on nitrogen conversion based on the microbial food web.

## 4. Conclusions

This study shows that paroxetine can have different effects on nitrogen transformation during different periods. In the early stage, paroxetine could promote complete nitrification by stimulating the Nitrospira and increase the accumulation of nitrate in sediment. During the later stage, paroxetine blocked the process of oxidizing ammonia to nitrate by inhibiting Nitrospira, and the nitrate content decreased. Paroxetine had no obvious effect on eukaryotic communities, but the interaction among trophic levels in the microbial food web could influence bacteria to carry out nitrogen transformation. The structural equation model proved that paroxetine can indirectly affect nitrogen-transforming bacteria through a top-down mechanism (i.e., predation) based on the microbial food webs, thus influencing nitrogen transformation. Therefore, attention should be paid to the ecological risk of paroxetine to microbial food webs in rivers. Further studies should focus on the combined effects of different types of antidepressants on aquatic ecosystems.

## Figures and Tables

**Table 1:** Comparison of α-diversity indices for eukaryotic communities under different times. C: the sediment with no paroxetine added; L: 10 μg/L paroxetine; M: 100 μg/L paroxetine; H: 1000 μg/L paroxetine.

Samples	Chao1 Index	ACE Index	Simpson Index	Shannon Index
C-6d	1303.455	1308.979	0.954122	6.399949
C-12d	1259.007	1273.765	0.927629	5.935353
C-18d	1649.173	1690.546	0.966728	6.623691
L-6d	1482.061	1479.052	0.982247	7.328378
L-12d	1200.021	1221.052	0.962134	6.803168
L-18d	1435.575	1347.239	0.968264	6.634653
M-6d	1887.182	1908.239	0.971867	7.001973
M-12d	1335.679	1328.988	0.947955	6.202316
M-18d	1427.407	1386.27	0.983062	7.209895
H-6d	2115.781	2197.906	0.948932	6.364666
H-12d	2003.015	1936.051	0.98843	7.652237
H-18d	1477.005	1521.179	0.953361	6.300156

## Supplementary Materials

The following supporting information can be downloaded at: https://www.mdpi.com/article/10.3390/ijerph192114602/s1, Figure S1. Relative abundance of metabolic pathways on KEGG categories (level 1). Figure S2. Heat map of relative abundance of eukaryotes in different levels of treatment groups on the 6th, 12th and 18th days. (a) is at class level and (b) is at order level; C: sediment without paroxetine addition; L: 10 μg/L paroxetine; M: 100 μg/L paroxetine; H: 1000 μg/L paroxetine. Table S1. Results of ANOVA for metabolism pathways in paroxetine treatment group. Table S2. Results of ANOVA for eukaryotic α-diversity at different concentrations and at different times. Table S3. Standardized total, direct and indirect effects of all the independent variables on dependent variables.
