# Supplementary material for: The Effects of Paroxetine on Benthic Microbial Food Web and Nitrogen Transformation in River Sediments
Source: Int J Environ Res Public Health. 2022 Nov 7;19(21):14602. doi: 10.3390/ijerph192114602 (PMC9657768; doi:10.3390/ijerph192114602)
Supplement: Supplementary file 1 [file ijerph-19-14602-s001.zip › ijerph-1959454-supplementary.pdf]

Supporting information

for

The effects of Paroxetine on benthic microbial food web and nitrogen transformation in river sediments

Yi Li[1], Xinqi Chen[1,2], Xinzi Wang[1], Jiahui Shang[1], Lihua Niu[1], Longfei Wang[1], Huanjun Zhang[1], Wenlong Zhang[1,] *

[1] Key Laboratory of Integrated Regulation and Resource Development of Shallow Lakes of Ministry of Education, College of Environment, Hohai University, Nanjing 210098, PR China

[2] Jiangsu Nanjing Environmental Monitoring Center, Nanjing 210013, PR China

* Corresponding author

Dr. Wenlong Zhang

Tel: 86-25-83787062

Fax: 86-25-83787062

E-mail: 1223zhangwenlong@163.com

This supplementary material contained 7 pages, 2 Figures, 3 Tables.

Figures and Tables captions:

Figure S1. Relative abundance of metabolic pathways on KEGG categories (level 1).

Figure S2. Heat map of relative abundance of eukaryotes in different levels of treatment groups on the 6th, 12th and 18th days. (a) is at class level and (b) is at order level; C: sediment without paroxetine addition; L: 10 μg/L paroxetine; M: 100 μg/L paroxetine; H: 1000 μg/L paroxetine.

Table S1. Results of ANOVA for metabolism pathways in paroxetine treatment group.

Table S2. Results of ANOVA for eukaryotic α-diversity at different concentrations and at different times.

Table S3. Standardized total, direct and indirect effects of all the independent variables on dependent variables.

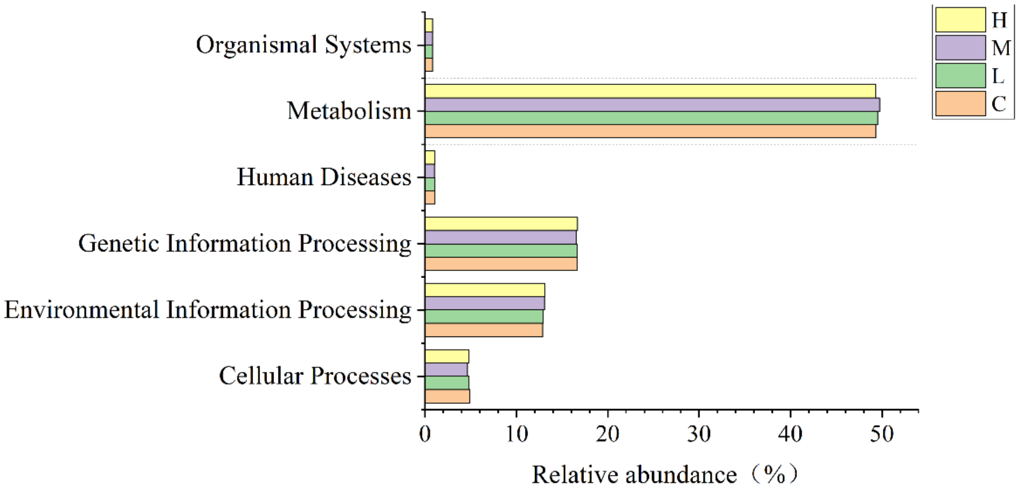

Figure S1. Relative abundance of metabolic pathways on KEGG categories (level 1).

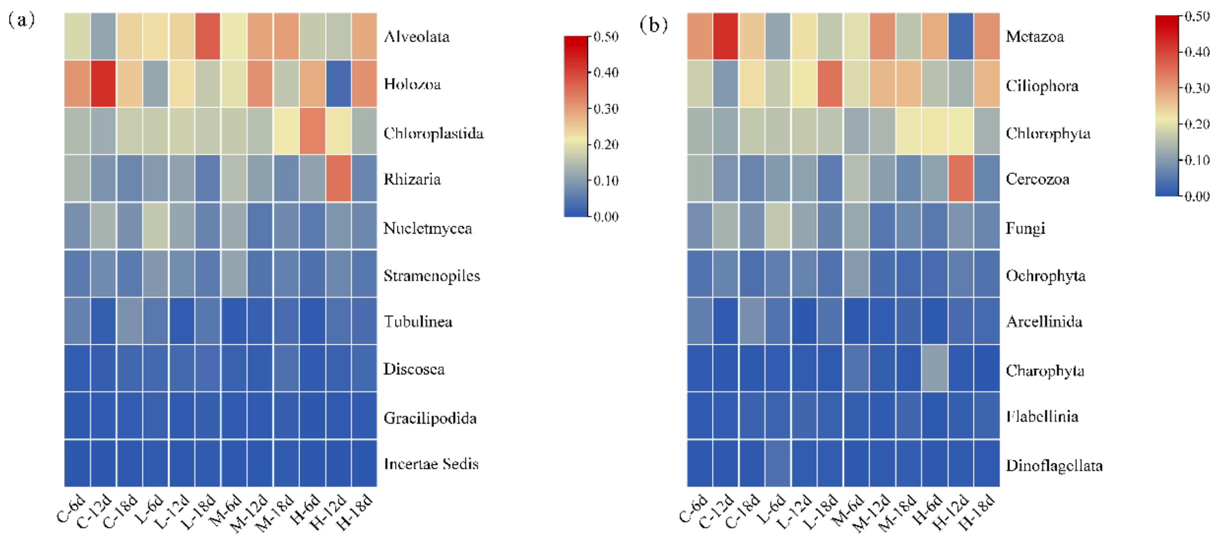

Figure S2. Heat map of relative abundance of eukaryotes in different levels of treatment groups on the 6th, 12th and 18th days. (a) is at class level and (b) is at order level; C: sediment without paroxetine addition; L: 10 μg/L paroxetine; M: 100 μg/L paroxetine; H: 1000 μg/L paroxetine.

Table S1. Results of ANOVA for metabolism pathways in paroxetine treatment group.

Metabolisms	P	F
Xenobiotics Biodegradation and Metabolism	0.151	5.165
Nucleotide Metabolism	0.068	13.241
Metabolism of Terpenoids and Polyketides	0.054	17.111
Metabolism of Other Amino Acids	0.141	5.610
Metabolism of Cofactors and Vitamins	0.058	15.670
Lipid Metabolism	0.048	19.317
Glycan Biosynthesis and Metabolism	0.062	14.627
Energy Metabolism	0.033	28.937
Carbohydrate Metabolism	0.019	51.529
Biosynthesis of Other Secondary Metabolites	0.010	97.722
Amino Acid Metabolism	0.057	16.203

Table S2. Results of ANOVA for eukaryotic α-diversity at different concentrations and at different times.

Factors	Chao 1 index		ACE index		Simpson index		Shannon index	
	F	P	F	P	F	P	F	P
Time	0.720	0.513	0.929	0.430	0.386	0.690	0.051	0.950
Concentration	2.238	0.161	2.337	0.150	0.823	0.517	0.751	0.552

Table S3. Standardized total, direct and indirect effects of all the independent variables on dependent variables.

		Meiofauna:Protozoa	Meiofauna:Bacteria	Ciliates:HF	Ciliates:Bacteria	HF:Bacteria	Amoeba:Bacteria
Standardized Total Effects	Meiofauna:Bacteria	0.896	0.000	0.000	0.000	0.000	0.000
	Ciliates:HF	-0.042	-1.248	0.000	0.000	0.000	0.000
	Ciliates: Bacteria	-0.53	1.181	-0.04	0.000	0.000	0.000
	HF: Bacteria	-0.046	0.655	-0.628	-0.297	0.000	0.000
	Amoeba: Bacteria	-0.578	0.365	0.136	0.449	-0.240	0.000
	NO^{2-}	0.446	0.153	-0.242	-0.144	-0.135	0.562
	NO^{3-}	0.099	-0.108	0.087	0	0	0.000
	NH_4^+	-0.115	-1.634	-0.399	0.096	0.314	-0.174
Standardized Direct Effects	Meiofauna:Bacteria	0.896	0.000	0.000	0.000	0.000	0.000
	Ciliates:HF	1.076	-1.248	0.000	0.000	0.000	0.000
	Ciliates: Bacteria	-1.545	1.131	-0.04	0.000	0.000	0.000
	HF: Bacteria	-0.418	0.209	-0.639	-0.297	0.000	0.000
	Amoeba: Bacteria	-0.456	0.075	0.000	0.378	-0.240	0.000
	NO^{2-}	0.546	0.000	-0.334	-0.397	0.000	0.562
	NO^{3-}	0.102	0.000	0.087	0.000	0.000	0.000
	NH_4^+	1.979	-2.293	-0.194	0.256	0.272	-0.174
Standardized Indirect Effects	Meiofauna:Bacteria	0.000	0.000	0.000	0.000	0.000	0.000
	Ciliates:HF	-1.118	0	0.000	0.000	0.000	0.000
	Ciliates: Bacteria	1.015	0.049	0.000	0.000	0.000	0.000
	HF: Bacteria	0.371	0.447	0.012	0.000	0.000	0.000
	Amoeba: Bacteria	-0.122	0.289	0.136	0.071	0.000	0.000
	NO^{2-}	-0.1	0.153	0.092	0.253	-0.135	0.000
	NO^{3-}	-0.004	-0.108	0	0	0	0.000
	NH_4^+	-2.094	0.659	-0.205	-0.159	0.042	0.000
